# Rapid inactivation of *Toxoplasma gondii* bradyzoites during formulation of dry cured ready-to-eat pork sausage

**DOI:** 10.1016/j.fawpar.2018.e00029

**Published:** 2018-08-24

**Authors:** D.E. Hill, J. Luchansky, A. Porto-Fett, H.R. Gamble, V.M. Fournet, D.S. Hawkins-Cooper, J.F. Urban, A.A. Gajadhar, R. Holley, V.K. Juneja, J.P. Dubey

**Affiliations:** a USDA, ARS, NEA, Animal Parasitic Diseases Laboratory, BARC-East, Bldgs. 1001 & 307-C, Beltsville, MD 20705, United States of America; b USDA, ARS, NEA, Food Safety and Intervention Technologies, 600 E. Mermaid Ln. ERRC, Wyndmoor, PA 19038-8598, United States of America; c National Academy of Sciences, 500 Fifth Street NW, Washington, DC 20001, United States of America; d USDA, ARS, BHNRC, Diet, Genomics, and Immunology Laboratory, BARC-East, Bldgs. 307-C, Beltsville, MD 20705, United States of America; e University of Saskatchewan, Department of Veterinary Microbiology, 52 Campus Drive, Saskatoon, SK S7N5B4, Canada; f University of Manitoba, Faculty of Agricultural and Food Sciences, Room 250 Ellis Building, 13 Freedman Crescent, Winnipeg, MB R3T 2N2, Canada

## Abstract

Curing processes for pork meat in the U.S. currently require individual validation of methods to demonstrate inactivation of *Trichinella spiralis*, a nematode parasite historically associated with pork. However, for protozoan parasites, no such strictures exist. It has been assumed, with little evidence, that curing processes required to inactivate *Trichinella* also inactivate *Toxoplasma gondii*. Currently no model of meat chemistry exists that can be correlated with inactivation of *T. gondii*. Given the possibility of the presence of *T. gondii* in pork meat, and the frequent use of pork for ready-to-eat (RTE) products not intended to be cooked, curing methods which inactivate *T. gondii* early in the curing process would be of great value to producers. In this study, we tested the effect of five variables – salt/brine concentration, water activity (a_w_), pH, temperature, and time on inactivation of *T. gondii* bradyzoites in tissue cysts using low and high endpoints for common curing treatments during preparation of dry cured pork sausage. Survival of *T. gondii* bradyzoites at each stage of preparation was assessed using a mouse bioassay. Results indicated that encysted *T. gondii* bradyzoites do not survive the early stages of the dry curing process within the endpoint parameters tested here, even at levels of NaCl that are lower than typically used for dry curing (1.3%). Exposure of *T. gondii* encysted bradyzoites to curing components in the formulated batter resulted in rapid inactivation of bradyzoites. These data suggest that the use of dry curing components may be effective for controlling *T. gondii* potentially transmitted through RTE meats, rendering them safe from risk with respect to *T. gondii* transmission to human consumers.

## Highlights

• We have investigated salt/brine concentration, water activity (α_w_), pH, temperature, and time- for their effect on inactivation of *Toxoplasma gondii* bradyzoites in tissue cysts in pork meat during the dry cure process used to make pepperoni sausage.
• NaCl at 1.3% or above is effective at rapidly inactivating bradyzoites within tissue cysts in ground pork during the fermentation process.
• The treatments reliably predict inactivation of *Toxoplasma gondii*, and can be extrapolated to various curing processes within the high and low endpoints tested to eliminate the need for individual ready-to-eat product validation.

## 1 Introduction

Though curing processes have been used for centuries as a method to preserve meat products in a state that renders them microbiologically safe for human consumption in the absence of cooking, no dry curing processes have been validated for inactivation of protozoan parasites found in meat, such as *Toxoplasma gondii*. In the U.S., 2.8% of market weight pigs at the farm level are infected with *T. gondii* (Hill et al., 2010), while the prevalence of *T. gondii* in fresh pork meat found in the meat case is >7 times lower, at 0.38% (Dubey et al., 2005). Many of the processes that lead to the inactivation of *T. gondii* bradyzoites in fresh meat products are unknown. However, pumping of fresh pork meat with NaCl and lactate solutions, which occurs in 50% of fresh pork products, has been shown to rapidly inactivate *T. gondii* bradyzoites contained within the pumped meat (Hill et al., 2004, Hill et al., 2006a). These data suggest that NaCl, which is the major additive in dry cured products, or combinations of NaCl and other components, are capable of inactivating bradyzoites in fresh pork. In this study, common curing methods were used to prepare a pepperoni style dry cured sausage with *T. gondii* infected pork meat, monitoring five common measurements during curing – salt/brine concentration, α_w_, pH, temperature, and time, and parasite inactivation during all phases of production until the endpoint water activity (α_w_) was reached after the drying phase. Using the newly developed inactivation data, existing and newly developed curing process that fall within the tested parameters can be assessed for risk mitigation with respect to *T. gondii* based on the meat chemistry of the final product. These data provide a baseline for the ultimate goal of developing a searchable *T. gondii* inactivation model which can be used to populate the USDA, ARS Pathogen Modeling Program (PMP; download at https://www.ars.usda.gov/northeast-area/wyndmoor-pa/eastern-regional-research-center/residue-chemistry-and-predictive-microbiology-research/docs/pathogen-modeling-program/pathogen-modeling-program-v ersion-70-installation/) and used by producers and regulators to assess the safety of new curing processes without the need for individual process validation.

## 2 Materials and methods

Time periods for analysis were developed in a pilot study, in which non–infected meat was fabricated as described below and samples taken for meat chemistry on a daily basis as previously described (Hill et al., 2017). Based on the speed of fermentation, time periods for sampling infected meat were determined, and the process adjusted to meet the criteria required for typical commercially prepared product. For experimental data collection, a total of 20 mixed breed pigs, 10–12 weeks of age each, were inoculated subcutaneously with 10,000 VEG strain *Toxoplasma gondii* bradyzoites freed from the tissue cyst. Tissue cysts were isolated from the brains of *T. gondii* infected mice inoculated (per os) 40 days previously with 50 VEG strain oocysts. Infected pigs were housed at the USDA's Beltsville Agricultural Research Center, and grown to near market size (67 to 75 kg). Infected pigs were housed in separate pens and quarantined from non-infected pigs in accordance with the Animal Welfare Act, Guide for the Care and Use of Laboratory Animals (https://www.nap.edu/search/?term=Guide+for+the+Care+and+Use+of+Laboratory+ Animals+) and with the approval of the USDA/ARS Beltsville Area Institutional Animal Care and Use Committee (BAACUC Approval # 15-014). After 60 days, infected pigs were humanely euthanized by electrocution followed by exsanguination. The triceps, picnics, hams, neck, rump, and loins were collected and hand trimmed of excess fat and connective tissue to yield a total of 23 to 30 kg of meat from each animal. Back fat was also collected from each animal and supplemented with purchased pork back fat (C & C Meats, Upper Marlboro, MD) for sausage preparation. Pepperoni chubs were prepared using the multivariate protocols described in Table 1, which are common commercial formulations.

**Table 1:**
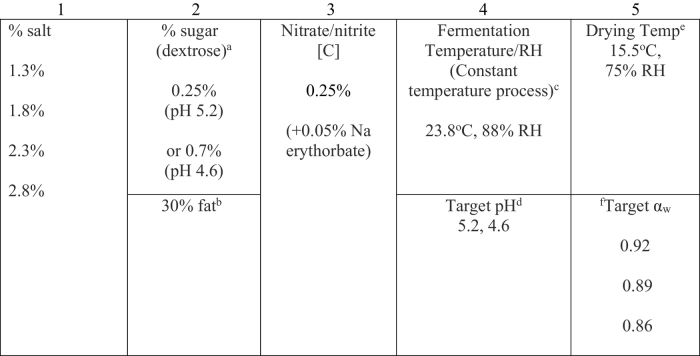
Curing Regimen for production of dry-cured sausage. Column 1: Four NaCl concentrations were used in batter formulations. Column 2^a^: Half of each preparation of salted batter were prepared with 0.7% dextrose, the other half was prepared with 0.25% dextrose. Added dextrose in the batter is fermented by added *Pediococcus acidilactici* and *Staphylococcus carnosus* lactobacillus, resulting in acidification of the batter matrix. The higher the dextrose concentration, the longer fermentation proceeds, resulting in a lower final pH. 2^b^: All batters were formulated with 30% pork back fat. Column 3: All batters were formulated with 0.25% sodium nitrate/nitrite curing salt as a preservative, and 0.05% sodium erythorbate to act as an antioxidant to preserve meat color and to accelerate the breakdown of sodium nitrate to nitrite by bacteria, speeding the curing process. Column 4^c^: All chubs were fermented at a constant temperature and RH of 23.8 °C and 88% RH. 4^d^: All chubs from each salt concentration prepared with 0.7% dextrose were fermented to pH 4.6, chubs prepared with 0.25% dextrose were fermented to pH 5.2. Column 5^e^: All chubs from each salt concentration and each pH were dried at 15.5 °C, 75% RH. 5^f^: Once chubs reached α_w_ of 0.92, 0.89, or 0.86, meat from each chub was digested and inoculated into mice (n = 5) to determine continued viability of *T. gondii* present in meat.

Approximately 100 kg of raw pepperoni batter was prepared in each of 8 batches. Pepperoni was prepared using *T. gondii* infected pork muscles described above and pork back fat to achieve a ratio of about 70% lean meat to 30% fat. The blend of pork meat and chilled fat was coarse ground using a sanitized mixer/grinder through an accessory grinding plate with 9.5 mm holes (Model 4346; Hobart Corporation, Troy, OH, USA), and mixed for 20 min at 4 °C to provide a uniform distribution of parasites. The mixer/grinder was sanitized with a quaternary ammonium-based germicide, multipurpose detergent (Misty® Biodet ND32), rinsed thoroughly for 3 min with hot water (87.8 °C), and then allowed to air dry at room temperature for up to 1 h. The coarse ground pork was re-loaded into the sanitized mixer/grinder, reground to produce to ≥3 mm particles, and dry ingredients were added to achieve variable combinations of salt (1.3, 1.8, 2.3, and 2.8%; high-grade sodium chloride, The Canadian Salt Company Ldt., Pointe Claire, QC, Canada), 0.25% (for pH endpoint 5.2) or 0.7% (for pH endpoint 4.6) dextrose/sugar to achieve common curing method endpoints for pH (Tate & Lyle of Nealanders International Inc., Mississauga, ON, Canada), and 0.25% cure (sodium nitrate/nitrite; Wiberg Canada Corporation, Oakville, ON, Canada). The pepperoni batter was mixed for about 1 min before the addition of starter culture mixture (*Pediococcus acidilactici* and *Staphylococcus carnosus*) in water (Formula 102; Trumark Inc., Linden, NJ, USA), per the manufacturer's instruction to yield about 6 to 7 log_10_ CFU/g, respectively.

The pepperoni batter was blended for an additional 10 min to assure uniform distribution of additives, fine ground through a 4.7 mm accessory grinding plate, then extruded into water-softened 55 mm diameter cellulose-based casings (Naturin R2; Weinheim, Germany) using either a stainless steel table-top manual piston stuffer (9 kg capacity; Model FD-9051200; F. Dick, Esslingen, Germany) or a floor model, hydraulic-driven piston stuffer (18.6 kg capacity; Model SC-50, Koch Equipment, Kansas City, MO, USA), to form pepperoni chubs 254 mm in length, and weighing 500 g each. After stuffing, the pepperoni chubs were hand tied with twine, transferred to a temperature- and humidity-controlled walk-in incubator (EJS Systems Inc., Changrin Falls, OH, USA) with an air flow of 1.0 to 1.5 m/s, and hung vertically on racks so that the individual chubs were not touching. The relative humidity (RH; 88%) and air temperature (23.8 °C) were controlled and constantly monitored during fermentation (and drying at 15.5 °C) using the Dynamist 2000 System and Partlow MRC5000 chart recorder (EJS Systems Inc., Changrin Falls, OH, USA). The pepperoni chubs were fermented using constant temperature, degree•hour (the time in hours at a particular temperature multiplied by the degrees in excess of 15.6 °C (the critical temperature at which *Staphylococcal* growth effectively begins), and relative humidity to achieve target pH values of 5.2 and 4.6 as outlined in Table 1. To determine pH during fermentation, at 3 h intervals, 50 g samples from individual chubs representing each treatment were homogenized with 100 ml of deionized water for 1 min in a stomacher blender (Stomacher 3500, Thomas Scientific, Swedesboro, NJ, USA), and the pH of each sample was measured by immersing the electrode of the pH meter in the sample homogenate (Mettler Toledo pH/ion meter, Model 235, Schwerzenbach, Switzerland). Once the targeted pH was achieved (pH 5.2 or 4.6; end of fermentation, 24 h at 23.8 °C, 88% RH, α_w_ 0.99–1.0), chubs were dried at 15.5 °C, 75% RH, until the target α_w_ endpoints were reached (0.92, 0.89, and 0.86 α_w_) using the Dynamist 2000 System described above. Water activity and pH in each chub was recorded at the time of sampling. The α_w_ of the chubs was measured by placing 20 g samples from individual chubs from each treatment into a sampling cup and analyzing using an electronic water activity meter (Model HP23AW, Rototronic, Hauppauge, NY, USA) calibrated to 80% RH. The pH of sampled chubs was measured as described above.

The presence of viable *T. gondii* tissue cysts in the initial meat and batter mixtures was determined by digestion of ten 50 g samples of the final ground meat preparation, and 50 g of each of the 8 batter mixtures in acidified pepsin for isolation of bradyzoites followed by subcutaneous inoculation of the digests into 6–8 week old female Swiss-Webster mice (meat digestion procedure described below). One ml of each of the 10 meat sample digests was inoculated into a single mouse, and 1 ml of each of the batter digests was inoculated into each of 5 mice. Mice were maintained for 40 days, then were euthanized. Brain squashes were prepared from each mouse; these were examined for tissue cysts to determine the presence of viable *T. gondii* in the initial meat and freshly prepared batters used to prepare the dry cured pork chubs. Mice were also tested serologically by ELISA using serum collected during necropsy using mouse specific reagents (Hill et al., 2006b).

Three whole chubs from each batter formulation were randomly removed from the drying chamber and sampled on a weekly basis beginning on day 1 (end of fermentation) for 8 weeks, for digestion to isolate bradyzoites for mouse bioassay to determine infectivity of *T. gondii* bradyzoites exposed to conditions of the curing and drying processes. Water activity of each chub was also tested on the day of sampling to monitor the approach of the drying endpoint. The final samples were collected and digested on day 60 post stuffing, at which point all samples had reached 0.86 α_w_. For digestion of meat samples, the method of Dubey (2010) was used. Briefly, 50 g of meat was collected from each of 3 chubs, the casing was removed, the meat was chopped by hand into <3 mm particles, and then mixed with 250 ml of warmed (37 °C) saline in a blender. To this homogenate was added 250 ml of warmed saline, pH 1.2, containing 0.5% pepsin, w/vol [National Standard Formulary 1:10,000; American Labs Inc., Omaha, NE], 1% NaCl, and 1.4% hydrochloric acid, vol/vol. The ground meat and digestion fluid were stirred on a magnetic stir plate at 37 °C for 1 h, then filtered through gauze to remove any undigested material. After centrifugation at 1200 ×*g* for 10 min, the supernatant was decanted, and the sediment was washed repeatedly by centrifugation in 1.2% sodium bicarbonate buffer with phenol red to neutralize the HCl. The entire neutralized sediment was suspended in 10 ml of saline containing 100 units of penicillin and 100 μg of streptomycin/ml (Gibco, Grand Island NY). One ml of the suspension was inoculated subcutaneously into each of 2 mice for each chub (3 replicates (A, B, C) for each formulation, totaling 6 mice for each batter formulation at each sampling). Forty days after inoculation, mice were euthanized by cervical dislocation and thoracotomy in accordance with the Guide for Animal Care and Use of Laboratory Animals (BARC IACUC approved protocol # 16-014).Brain squashes were prepared from each mouse and were examined for the presence of tissue cysts to determine the continued presence of viable *T. gondii* in the dry cured pork chub used for the mouse inoculation, and anti-*Toxoplasma* ELISA was also performed to interrogate each mouse serum for antibody to *T. gondii*.

A cut-off of zero tissue cysts recovered from all 6 inoculated mice on all successive days (up to 8 weeks) was the criterion for determining the date of complete inactivation. A *t*-test was used to determine whether the decrease in viable *T. gondii* between meat and Day 1 (post fermentation) was significant. Regressions were used on each pH and NaCl combination to estimate the decrease in viable *T. gondii* over hours. A cut-off of 0.92, 0.89, and 0.86 a_w_ on all 3 sampled chubs was used to determine the end point of drying days. A linear regression was estimated with pH, NaCl, and their interaction as independent variables and the number of fermentation hours as the dependent variable.

## 3 Results

Initial water activity (a_w_) and pH in the ground meat was 0.99 and 6.94, respectively. Ten mice were inoculated with 1 ml of digest material from ten 50 g samples of the freshly ground and mixed meat (pre-cured) selected for preparation of the pepperoni batter; all 10 of these mice had *T. gondii* tissue cysts observed microscopically in brain squashes and were serologically positive for *T. gondii* infection by ELISA. Eight pepperoni batters containing the salt, sugar, nitrate, fermentation bacteria, and spice formulations described above was prepared immediately after the meat was ground. Water activity in the mixed batters is described in Table 2; immediately prior to fermentation, the a_w_ of the sausage batters was between a_w_ 0.96 and 0.98. Five mice each were inoculated with 1 ml of digest material from 50 g samples of the 8 pepperoni batter formulations; all 5 mice were positive in 2 of the formulations; 4 of 5 mice were positive in 5 of the formulations; and 3 of 5 mice were positive in 1 formulation for *T. gondii* tissue cysts observed microscopically in brain squashes and by ELISA (Table 2). Pepperoni batters were stuffed into 55 mm casings and fermented for 24 h until the final endpoint pH (5.2 or 4.6) was reached; water activity in all chubs was 0.965 or greater. Three chubs from each batter preparation were removed from the fermentation chamber at 3 h intervals during fermentation and 50 g from each chub were digested as described above. Five mice each were inoculated with 1 ml of digest material from 50 g samples of the 8 pepperoni batter formulations at 3 h intervals during the fermentation process; 2 mice were positive in 1 of the formulations (1.3% NaCl, pH 4.6 endpoint) for *T. gondii* tissue cysts observed microscopically in brain squashes and by ELISA at the first 3 h interval; all subsequent tests for *T. gondii* tissue cysts in brain squashes and ELISA were negative in all batter formulations and at all 3 h intervals (Table 3). At the end of fermentation, all chubs from all batter formulations were dried as described above to final endpoint a_w_ of 0.92, 0.89, and 0.86, and digests were inoculated into mice as described above. At necropsy, all mice were negative for tissue cysts in the brain and by ELISA serology for *T. gondii*.

**Table 2:** Pre-fermentation batter meat chemistry and observed tissue cysts in brain squashes/ELISA OD in batter inoculated mice, 40 days PI. Starting meat from *T. gondii* infected pigs used for preparation of pepperoni chubs was bioassayed in mice for infectivity after harvesting, and prior to addition of any curing additives. Starting α_w_ and pH were 0.99 and 6.94, respectively; typical α_w_ of fresh meat is at or near 1.0, and pH is at or near 7.0. Ten 50 g samples were digested as described, and inoculated s.c. into 10 Swiss-Webster mice. After 40 days PI, all ten mice were tissue (brain cysts observed) and serologically positive (ELISA OD > 0.30) for *T. gondii*. Immediately after curing additives were mixed into the starting meat, and before the beginning of fermentation, 50 g samples were taken from each of 8 batter formulations whose pH endpoints after fermentation were intended to be pH 4.6 or 5.2 (NaCl concentrations of 1.3–2.8, and dextrose concentrations of 0.7 or 0.25%, respectively). Water activity was between 0.96 and 0.98, and pH was between 6.52 and 6.72, slightly lower than the starting meat as a result of the addition of water-binding salt and curing additives. Three to 5 mice were tissue (brain cysts observed) and serologically positive (ELISA OD > 0.30) for *T. gondii* after inoculation with digests from each batter preparation. No discordant results were observed (tissue positive/ELISA negative, or ELISA positive/tissue negative).

Starting meat^a^	[NaCl]	α_w_	pH	Brain cyst/ELISA OD	Mice inoculated, n = 10			Brain cyst/ELISA OD
					Brain cyst/ELISA OD	Brain cyst/ELISA OD	Brain cyst/ELISA OD	
	N/A	0.99	6.94	pos/0.385	pos/0.309	pos/0.454	pos/0.428	pos/0.731
				pos/0.309	pos/1.462	pos/0.578	pos/0.798	pos/0.603
Batter Formulation					Mice inoculated, n = 5			
pH endpoint 4.6				Mouse #1	Mouse #2	Mouse #3	Mouse #4	Mouse #5
	1.3	0.96	6.58	pos/0.603	pos/0.385	pos/0.454	pos/0.578	neg/0.128
	1.8	0.97	6.72	pos/0.798	pos/0.731	pos/1.462	neg/0.173	pos/0.442
	2.3	0.96	6.56	pos/0.658	pos/0.485	neg/0.111	pos/0.472	pos/0.335
	2.8	0.98	6.52	pos/0.309	pos/1.414	pos/0.409	pos/0.557	pos/0.990
pH endpoint 5.2								
	1.3	0.98	6.61	pos/0.424	neg/0.157	pos/0.258	pos/0.265	neg/0.178
	1.8	0.96	6.58	neg/0.162	pos/0.308	pos/0.539	pos/0.475	pos/0.438
	2.3	0.97	6.56	pos/1.545	pos/0.604	pos/1.665	pos/1.522	pos/1.434
	2.8	0.97	6.54	pos/0.801	neg/0.10	pos/0.355	pos/0.423	pos/0.571

a After harvest and grinding, no additives.

**Table 3:** Meat chemistry during timed fermentation. During fermentation, pH values dropped rapidly and reached pH 5.2 by 9 h and pH 4.6 by 15 h post initiation of fermentation. Water activity remained stable as expected since fermentation is accomplished at 88% relative humidity.

Fermentation time (in hours)	0	3	6	9	12	15
Batter formulations	pH/αw	pH/αw	pH/αw	pH/αw	pH/αw	pH/αw
4.6–1.3%	5.61/0.966^a^	5.59/0.956^b^	5.32/0.952	5.06/0.962	4.77/0.965	4.59/0.940
4.6–1.8%	5.61/0.961	5.41/0.956	5.40/0.950	5.14/0.954	4.84/0.957	4.63/0.964
4.6–2.3%	5.58/0.955	5.51/0.954	5.42/0.953	5.27/0.946	4.89/0.952	4.59/0.987
4.6–2.8%	5.57/0.955	5.59/0.951	5.48/0.946	5.24/0.947	4.88/0.942	4.60/0.964
5.2–1.3%	5.62/0.955	5.60/0.959	5.35/0.951	5.19/0.967		
5.2–1.8%	5.58/0.960	5.65/0.949	5.30/0.954	5.21/0.955		
5.2–2.3%	5.62/0.958	5.66/0.951	5.44/0.944	5.14/0.953		
5.2–2.8%	5.61/0.959	5.67/0.945	5.49/0.941	5.17/0.947		

a Mean pH and water activity from 3 chubs taken from each batter at 3 h intervals during fermentation.

b 2/5 mice from 1 chub were *Toxoplasma* positive by observation of tissue cysts in brain squashes and by ELISA; All other inoculated mice were negative.

The number of mice positive for *T. gondii* tissue cysts was significantly lower in mice inoculated with digests from chubs fermented for <6 h than in mice inoculated with digests from the original meat or batter preparations (all *p* < 0.05; Table 2, Table 3), and was reduced 100% after 6 h of fermentation. The pH of each chub was checked at the time of collection. The pH remained stable after fermentation over the course of the 60 day collection period; endpoint 4.6 chubs ranged between pH 4.51 and 4.62, while the endpoint 5.2 chubs ranged between 5.10 and 5.25. Both the main effects of pH and NaCl significantly affected the number of days until a_w_ reached 0.92 (*F*_1,4_ = [32.9, 55.6]; *p* = [0.005, 0.002], respectively; 0.89 (*F*_1,4_ = [37.8, 55.6]; *p* = [0.005, 0.002]; and 0.86 (*F*_1,4_ = [45.9, 55.6]; *p* = [0.005, 0.002], Table 3). Fermentation to pH 5.2 was accomplished in 10 h post stuffing, while pH 4.6 was reached in 16 h post stuffing using the conditions described. Inactivation of *T. gondii* was accomplished well before fermentation to the endpoint pH was complete. After 6 h of fermentation, inactivation was complete, however, the pH in sampled chubs had reached only 5.73 in the endpoint pH 4.6 batters, and 5.71 in the endpoint pH 5.2 batters.

A summary of the results for complete *T. gondii* inactivation in dry cured sausage indicates the following proposed rule is applicable:

If pH is >4.6 and ≤ 5.2, and % NaCl ≥1.3%, 6 h of fermentation is required to achieve complete inactivation of *T. gondii* bradyzoites (in tissue cysts). This proposed rule is not valid for pH outside of the range (4.6, 5.2), nor for % NaCl <1.3.

## 4 Discussion

In the U.S., *T. gondii* infection in humans is thought to commonly result from ingestion of tissue cysts contained in under-cooked meat (Dubey and Beattie, 1988; Roghmann et al., 1999; Lopez et al., 2000), though the exact contribution of food-borne toxoplasmosis versus oocyst-induced toxoplasmosis to human infection is currently unknown. Hill et al. (2011) described a sporozoite specific antigen that correctly identified individuals infected with *T. gondii* via oocysts versus tissue cysts; application of this test to human sera and saliva from different geographic regions suggests that oocyst transmission may be responsible for 50–75% of human *T. gondii* infections, leaving 25–50% that may be foodborne (Muñoz-Zanzi et al., 2010; Mangiavacchi et al., 2016). *Toxoplasma gondii* infection is common in many animals used for food, including pigs, sheep, and goats. Viable *T. gondii* tissue cysts were isolated from 17% of 1000 adult pigs (sows) from a slaughter plant in Iowa (Dubey et al., 1995), and in 51 of 55 market weight pigs from New England, USA (Dubey et al., 2002). Serological surveys of pigs from Illinois pig farms indicated an infection rate of about 3.1% in market weight animals and 20.8% for breeding pigs, suggesting that age is a factor for pigs acquiring *T. gondii* infection (Weigel et al., 1995). Serological surveys of pigs on New England farms revealed an overall infection rate of 47% (Gamble et al., 1999). More recent surveys using NAHMS sera in the U.S. have documented seroprevalence rates of 2.8% in market pigs and 6% in sows (Hill et al., 2010). Virtually all-edible portions of an animal can harbor viable *T. gondii* tissue cysts, and tissue cysts can survive in food animals for years (Dubey et al., 1986). In the U.S., meat products containing pork are likely the most common meatborne source of the parasite for humans, however, the contribution of undercooked pork in RTE meats to *T. gondii* infection in humans is unknown. There are no inactivation protocols for *T. gondii* currently available for RTE meat products; all published protocols for inactivation of *T. gondii* involve either cooking or freezing of fresh meat (Dubey, 1988; Dubey et al., 1990). Currently there are no regulatory requirements for inactivation of *T. gondii* in meats, though some European food safety and public health governmental organizations have established frameworks for considering such regulations (Scandrett, 2015). The dearth of validated procedures for inactivation of *T. gondii* in RTE meat products limits producers and regulators in expanding product choices and modifying products to meet changing consumer expectations. An understanding of parameters of meat chemistry which can be used to predict inactivation of *T. gondii* during curing would provide a valuable resource for producers to meet safety requirements. Currently, no general model exists for inactivation of *T. gondii* which is readily accessible to producers and provides validated means to mitigate the risk of their products.

In this study, we have identified parameters for inactivation of *T. gondii* using a model curing process; these curing parameters fall within a range of processes which are commonly used by the pork industry for a variety of different dry cured RTE products. *T. gondii* viability studies are generally accomplished using mouse bioassay. Digestion of meat samples in pepsin was used to isolate and concentrate *T. gondii* tissue cysts and bradyzoites for detection by bioassay (Dubey, 1988; Dubey et al., 1990). The pepsin digestion process ruptured the *T. gondii* tissue cyst wall, releasing hundreds or thousands of bradyzoites. The bradyzoites can survive in the digest fluid for several hours, but are difficult to visualize microscopically due to debris from the digest in the sediment. Therefore, the digested material was bioassayed in mice (Dubey and Beattie, 1988; Dubey 2010). The mice inoculated with digested material were kept for 40 days before necropsy to detect *T. gondi*i tissue cysts in brain tissues and by ELISA.

It is important in food safety studies to demonstrate continuing infectivity of the *T. gondii* bradyzoites contained within tissue cysts as has been done here. It is not possible to visually determine whether tissue cysts contain viable parasites; only transmission studies in animals can determine continued viability. *Toxoplasma gondii* were recovered from mice inoculated with the starting meat and batter with no diminution of infectivity, while the fermentation procedure rapidly eliminated infectivity of bradyzoites, eliminating viability within 6 h after the start of fermentation. Results suggested that infectivity was eliminated in all of the fermenting meat matrices (chubs fermenting to pH 5.2 and pH 4.6, %NaCl 1.3–2.8) before fermentation was completed. This rapid inactivation suggests that a_w_ played a minimal role in the inactivation, since the level of unbound water in the chubs did not change significantly over the 16 h fermentation process (χ batter a_w_ = 0.97; χ Day 1 a_w_ = 0.965), and remained above 0.96 in all of the 8 pH/salt formulations up to the time of complete inactivation of bradyzoites (Table 2). Typical endpoint a_w_ for fermented, dry cured sausage such as pepperoni is 0.85 to 0.91 (Getty, 2005). Since temperatures were held constant at 23.8 °C in all samples during fermentation, the optimal temperature for activity of the fermentation bacteria and typical for the production of a dry cured pepperoni, the impact of the fermentation temperature alone on *T. gondii* infectivity in the batter mixtures could not be determined. Dubey et al. (1990) reported a time and temperature dependent inactivation of *T. gondii*; the lowest time and temperature combination tested which resulted in inactivation of bradyzoites was 52 °C for 9.5 min. Given that 23.8 °C is below the normal regulated body temperature of typical living mammalian hosts of *T. gondii*, and that *T. gondii* can survive for days in muscle of dead hosts at unregulated temperatures, it is unlikely that this temperature alone would result in inactivation.

The pH of pork meat immediately after harvesting from an animal is typically 7.0–7.2, and may drop to pH 5.5 in <24 h after slaughter due to formation of lactic acid (Lonergan, 2008). These data suggest that the drop to pH 5.7 at the time of complete inactivation after 6 h of fermentation played a minimal role in *T. gondii* inactivation during fermentation, since bradyzoites in tissue cysts in meat can survive for at least a week in tissues of dead animals as stated above. Additionally, Pott et al. (2013) described tissue cysts as capable of surviving for up to 26 days in environments at pH 5.0, and interestingly described tissue cysts as highly sensitive to salt concentration.

In the current study, the concentration of NaCl may be the most significant contributor to *T. gondii* inactivation. Regardless of pH, fermentation temperature, or water activity, *T. gondii* bradyzoites were inactivated in NaCl concentration of 1.3% or greater by 6 h after the beginning of fermentation. These data are similar to those found in Hill et al., 2004, Hill et al., 2006a which identified NaCl, potassium lactate, or sodium lactate concentrations of 1.4% or greater as effective in inactivating *T. gondii* bradyzoites in tissue cysts in fresh pork.

In conclusion, this study demonstrates that RTE pork sausage, using protocols as described here, can be rendered safe for *T. gondii* infection based upon careful monitoring of salt concentration and time. The specific conditions of NaCl concentration, pH and time, as shown in this study, could be used as a baseline to study and/or validate other curing methods, and to begin construction of a generalized model for inactivation of *T. gondii* in RTE pork. The USDA Pathogen Modeling Program (PMP) is a potential vehicle for constructing and facilitating dissemination of such a model. The PMP is a package of models that can be used to predict the growth and inactivation of foodborne bacteria, and are specific to certain bacterial strains and the specific environments (e.g., culture media, food matrix, etc.) that are used to generate the models (Hill et al., 2017). Since the objectives here were limited to defining relevant parameters, additional data should be generated based on the current findings for development of a complete model of inactivation of *T. gondii*.

## Conflict of interest statement

None of the authors have any financial or personal relationships that could inappropriately influence or bias the content of this paper.

This work was funded by the National Pork Board (project # 14-134).
